# Subtypes and symptoms of fecal incontinence in the Dutch population: a cross-sectional study

**DOI:** 10.1007/s00384-018-3051-5

**Published:** 2018-04-28

**Authors:** Maxime M. van Meegdenburg, Rob J. Meinds, Monika Trzpis, Paul M. A. Broens

**Affiliations:** 1 Department of Surgery, Anorectal Physiology Laboratory, University Medical Center Groningen, University of Groningen, PO Box 30 001, 9700 RB Groningen, the Netherlands; 2 Department of Surgery, Division of Pediatric Surgery, University of Groningen, University Medical Center Groningen, Groningen, the Netherlands

**Keywords:** Anorectal disorder, Population characteristics, Rectal diseases, Accidental bowel leakage

## Abstract

**Purpose:**

To study the distribution of subtypes and symptoms of fecal incontinence in the general Dutch population.

**Methods:**

We performed a cross-sectional study in a representative sample of the general Dutch population. All respondents (*N* = 1259) completed the Groningen Defecation and Fecal Continence questionnaire. We assigned the respondents to a so-called healthy subgroup (*n* = 1008) and a comorbidity subgroup (*n* = 251). The latter subgroup comprised the respondents who reportedly suffered from chronic diseases and who had undergone surgery known to influence fecal continence. We defined fecal incontinence according to the Rome IV criteria.

**Results:**

The combination of urge fecal incontinence and soiling was the most frequent form of fecal incontinence in the total study group, the “healthy” subgroup, and the comorbidity subgroup (49.0, 47.3, and 51.5%). Passive fecal incontinence was the least frequent form of fecal incontinence in all three groups (4.0, 5.4, and 2.2%). The prevalence and severity of fecal incontinence was significantly higher in the comorbidity subgroup than in the “healthy” subgroup. Only in the comorbidity subgroup did the fecally incontinent respondents feel urge sensation significantly less often before defecating than their fecally continent counterparts (16.5 versus 48.8%, *P* < 0.001).

**Conclusion:**

Urge fecal incontinence combined with soiling is commonest in the general Dutch population. Chronic diseases and bowel and pelvic surgery both increase and aggravate fecal incontinence.

**Electronic supplementary material:**

The online version of this article (10.1007/s00384-018-3051-5) contains supplementary material, which is available to authorized users.

## Introduction

Fecal incontinence (FI) can be a devastating disease and it may have a significant impact on people’s quality of life and healthcare costs. [1–4] Generally, FI is defined as involuntary loss of feces at least once a month. Different subtypes of FI are recognized and include the type of leakage (for example, soiling, urge FI, passive FI, or combined FI) [5–7]. In a recent systematic review, Ng and colleagues reported that the median prevalence of FI in representative samples of the general populations of amongst others Australia, New Zealand, the USA, the UK, and Canada is 7.3% and ranges from 2.0 to 13.2% [8]. These findings are in accordance with our study that showed a 7.9% prevalence of FI in the Netherlands [9]. To date, only a few studies investigated the prevalence of the subtypes of FI, all with varying results [10–12].

Variability in the prevalence of FI can be explained by several factors. Most important are the different diagnostic criteria for FI and its subtypes. The heterogeneity of data collection methods are also known to influence the prevalence of FI. For example, data were collected by questionnaires, telephonic interviews, and digital surveys [8]. Moreover, most of the studies on the prevalence of the subtypes of FI were performed in patient populations rather than in a sample representing the general population. Finally, the heterogeneity in demographic characteristics might influence the prevalence of FI, because age is considered to have a strong influence on the prevalence of FI [4, 8]. The higher prevalence of FI in the elderly in comparison to younger age groups may be caused by an increase in comorbidities, such as impaired mobility, which makes it more difficult to reach the toilet in time.

Our primary objective therefore was to examine the prevalence of the subtypes of FI in the general Dutch population. Secondarily, we aimed to investigate the distribution of the symptoms of FI. We also studied the influence on the distribution of the subtypes and symptoms of FI of somatic disorders and types of surgery that known to affect fecal continence.

## Materials and methods

### Study design

Between 1 September and 1 November 2015, we examined a cross section of the adult Dutch population using the Groningen Defecation and Fecal Continence (DeFeC) questionnaire (supplemental file) [13]. Data from the general Dutch population were collected by Survey Sampling International. This company, based in Rotterdam, the Netherlands, specializes in conducting surveys. They drew a population-based sample from their database and these people were sent a link that enabled them to fill out the DeFeC questionnaire digitally. Of the 3031 respondents who started filling out the questionnaire, 1642 (54.2%) filled it out completely. From the completed questionnaires, Survey Sampling International randomly selected 1259 (76.7%) respondents to obtain a representative cohort that was equally distributed across sex, region, and age in accordance with the population pyramid of the Netherlands (supplied by Statistics Netherlands) [14]. These 1259 respondents constituted our total study group of the general Dutch population.

Besides, we assigned each of the respondents in the total study group to either a “healthy” subgroup or a comorbidity subgroup. The comorbidity subgroup comprised 251 respondents who reported a history of bowel or pelvic surgery or who suffered from somatic diseases that could have influenced fecal continence, including intestinal resection, perianal fistula, anal sphincter surgery, hemorrhoid, prostate or rectal prolapse surgery, inflammatory bowel diseases, diabetes, cerebral stroke, neurological disorders (for example, spinal cord injury or multiple sclerosis), slow transit constipation, or congenital disorders (for example, anorectal malformation, Hirschsprung’s disease, sacrococcygeal teratoma, or spina bifida). The “healthy” subgroup comprised the remaining 1008 respondents.

### Definitions of demographic characteristics

A respondent’s highest level of education was classified as primary (primary or middle school), secondary (high school or vocational education), or tertiary (university or college). Respondents who lived in a small village or a city with a maximum of 50,000 inhabitants were classified as living in a rural environment, while respondents who lived in a city of more than 50,000 inhabitants were classified as living in an urban environment. Respondents’ body mass indices (BMIs, kg/m^2^) were classified according to WHO guidelines: underweight (< 18.5 kg/m^2^), normal weight (18.5 to 25 kg/m^2^), overweight (25 to 30 kg/m^2^), or obese (> 30 kg/m^2^).

### Definitions of defecation disorders

We defined FI according to the Rome IV criteria, namely recurrent uncontrolled passage of fecal material at least several times a month for the past 6 months [15]. In both the “healthy” subgroup and the comorbidity subgroup, we classified the respondents with FI according to the type of FI they had. Soiling was defined as accidental passage of small amounts of feces (that is, staining or soiling of underpants). Urge FI was defined as feeling a strong urge to defecate and being unable to reach the toilet in time to prevent FI, having to rush to the toilet to prevent FI, or the inability to postpone defecation for more than 5 min after feeling urge sensation. Passive FI was defined as accidental passage of large amounts of solid stool in the absence of urge sensation. To determine the severity of FI, we calculated the Vaizey incontinence score, [16] and the Continence Grading Scale as described by Jorge and Wexner [17].

All medical information was reported by the respondents themselves and because they filled in the questionnaires anonymously we could not review their medical records.

### Statistical analysis

Data were analyzed with SPSS for Windows, Version 23.0 (IBM SPSS Statistics, IBM Corporation, Armonk, NY). We reported median, minimum, and maximum values. Comparisons between the “healthy” subgroup and the comorbidity subgroup were performed using the Fisher exact and the Mann-Whitney tests. Statistical significance was defined as *P* ≤ 0.05.

## Results

The total study group comprised 1259 respondents with a median age of 49 years (range 18 to 85 years). The respondents in the “healthy” subgroup were significantly younger than the respondents in the comorbidity subgroup (48 years versus 57 years, *P* < 0.001) and it consisted of significantly more women than the comorbidity subgroup (55.8 versus 47.0%, *P* = 0.013). Other demographic characteristics of the included respondents are presented in Table 1.

**Table 1:** Respondent characteristics

	Total study group *N* (%)	“Healthy” subgroup *n* (%)	*P*	Comorbidity subgroup *n* (%)
Overall	1259 (100)	1008 (100)		251 (100)
Sex			0.013	
Men	579 (46.0)	446 (44.2)		133 (53.0)
Women	680 (54.0)	562 (55.8)		118 (47.0)
Educational level			0.196	
Primary	260 (20.7)	198 (19.6)		62 (24.7)
Secondary	505 (40.1)	407 (40.3)		98 (39.0)
Tertiary	494 (39.2)	403 (40.0)		91 (36.3)
Residence			0.711	
Rural	436 (34.6)	352 (34.9)		84 (33.5)
Urban	823 (65.4)	656 (65.1)		167 (66.5)
Body mass index			< 0 .001	
Underweight	26 (2.1)	24 (2.4)		2 (0.8)
Normal weight	549 (43.6)	463 (45.9)		86 (34.3)
Overweight	423 (33.6)	331 (32.8)		92 (36.7)
Obese	261 (20.7)	190 (18.8)		71 (28.3)

### Distribution of subtypes of FI and severity of FI

**Fig. 1:**
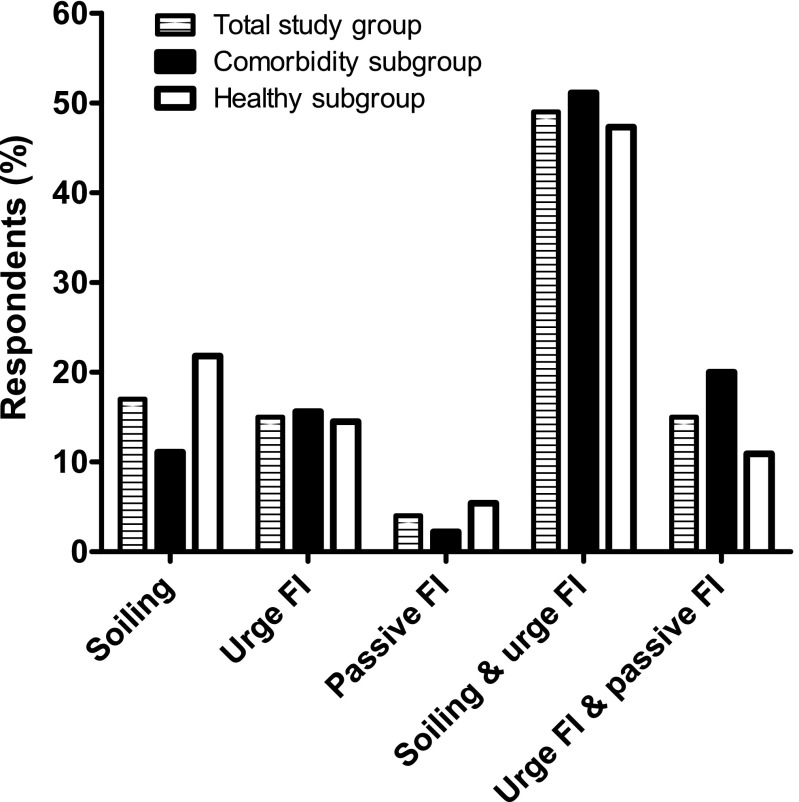
The subtypes of fecal incontinence (FI) in the total study group, the comorbidity subgroup, and the “healthy” subgroup. In the total study group and in the “healthy” subgroup, most respondents suffered from the combination of soiling and urge FI (49.0 and 47.3%), followed by soiling alone (17.0 and 21.8%), urge FI alone (15.0 and 14.5%), the combination of urge FI and passive FI (15.0 and 10.9%), and passive FI alone (4.0 and 5.4%). In the comorbidity subgroup, most respondents suffered from the combination of soiling and urge FI (51.1%), followed by the combination of urge FI and passive FI (20.0%), urge FI alone (15.6%), soiling alone (11.1%), and passive FI alone (2.2%)

In the total study group, the prevalence of FI was 7.9%. The prevalence of FI was significantly lower in the “healthy” subgroup than in the comorbidity subgroup (5.5 versus 17.9%, *P* < 0.001). As depicted in Fig. 1, the combination of soiling and urge FI was most common in the total study group, followed by the “healthy” subgroup and the comorbidity subgroup (49.0, 47.3, and 51.1%). Least common in all three groups was passive FI (4.0, 5.4, and 2.2%, respectively).

**Fig. 2:**
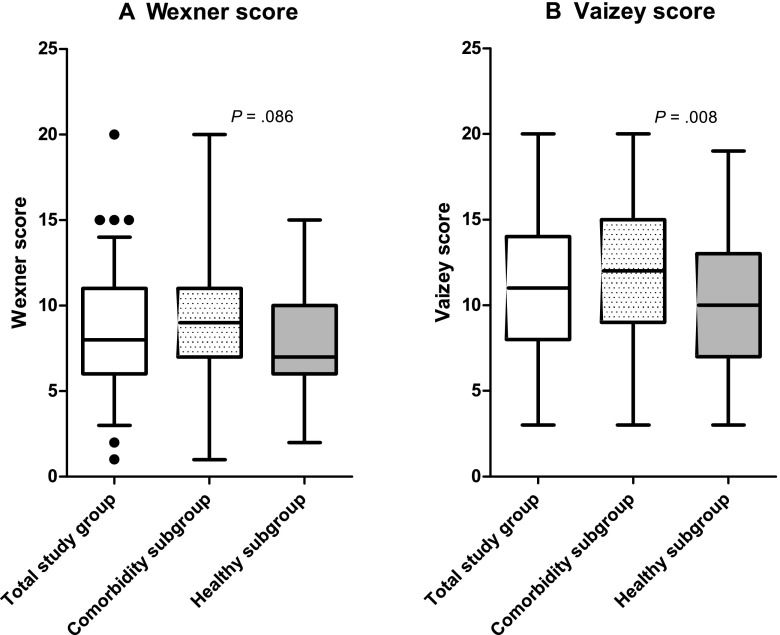
The Wexner and Vaizey scores in the total study group, the comorbidity subgroup, and the “healthy” subgroup. **a** The median Wexner score was 8 (range 1 to 20) in the total group and it tended to be lower in the “healthy” subgroup compared to the comorbidity subgroup (7 versus 9, *P* = 0.086). The black dots in the total study group represent outliers. **b** The median Vaizey score was 11.0 (range 3 to 20) in the total group and was significantly lower in the ‘healthy’ subgroup than in the comorbidity subgroup (10 versus 12, *P* = 0.008)

The median Wexner score and the median Vaizey score in the total study group with FI was 8 (range 1 to 20) and 11 (range 3 to 20), respectively. As illustrated in Fig. 2, the median Wexner and Vaizey scores were higher in the fecally incontinent respondents in the comorbidity subgroup than in the fecally incontinent respondents in the “healthy” subgroup (9 versus 7, *P* = 0.086 and 12 versus 10, *P* = 0.008). In all three groups, the Wexner and Vaizey scores did not differ significantly as regards sex or age (data not shown).

### Characteristics of FI

**Fig. 3:**
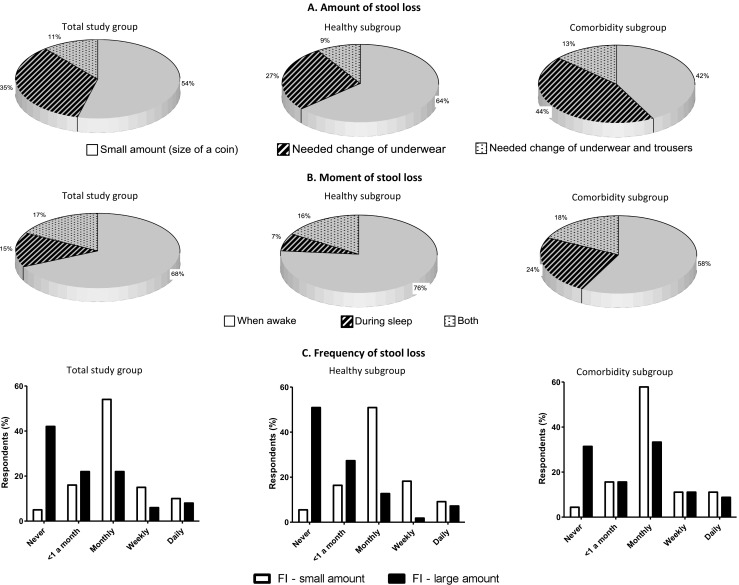
The amount of stool lost, the moment of stool loss, and the frequency of fecal incontinence (FI) in the total study group, the comorbidity subgroup, and the “healthy” subgroup. **a** While most respondents in the total study group and “healthy” subgroup lost a negligible amount of stool, most respondents in the comorbidity subgroup lost an amount that necessitated a change of underwear. In all three groups, only a few respondents lost a larger amount of stool that necessitated a change of outerwear. **b** In all three groups, most respondents only suffered from FI while they were awake. Respondents in the comorbidity subgroup suffered from FI more often while awake and during sleep compared to the other two groups. **c** In all three groups, most respondents lost small amounts of stool accidentally one to three times a month. Respondents who lost large amounts of stool, most often reported this to be less than once a month up to several times a month

We also asked the FI respondents about the amount of stool loss, whether stool was lost while awake or during sleep, and the frequency of stool loss. Most of the respondents with FI in the total group and “healthy” subgroup lost small amounts of stool, while most of the respondents with FI in the comorbidity subgroup needed to change their underwear (54, 64, and 44%, Fig. 3a). In all three groups, stool was most often lost while awake (68, 76, and 58%, Fig. 3b). Respondents in the comorbidity subgroup suffered from FI while asleep significantly more often than did respondents in the “healthy” subgroup (24 versus 7% *P* = 0.048). Lastly, loss of small amounts of stool one to three times a month was seen in both groups, while the loss of larger amounts of stool was less frequent (Fig. 3c).

### Distribution of symptoms associated with FI and use of medication

First, we analyzed whether the respondents with FI used stool softeners (laxatives) or stool hardeners (antidiarrheals, Table 2). In all three groups, we found that respondents with FI used laxatives and antidiarrheals significantly more often than respondents without FI.

**Table 2:** Use of medication influencing FI and symptoms of FI in the “healthy” and comorbidity subgroups

	Total study group			“Healthy” subgroup			Comorbidity subgroup		
	No FI *n* (%)	FI *n* (%)	*P*	No FI *n* (%)	FI *n* (%)	*P*	No FI *n* (%)	FI *n* (%)	*P*
Used laxative medication	64 (5.5)	27 (27.0)	< 0.001	49 (5.1)	10 (18.2)	< 0.001	28 (13.6)	19 (42.2)	< 0.001
Used antidiarrheals medication	8 (0.7)	15 (15.0)	< 0.001	6 (0.6)	4 (7.3)	0.013	2 (1.0)	13 (28.9)	< 0.001
Felt urge before defecating			0.002			0.329			< 0.001
Yes	887 (76.5)	61 (61.0)		715 (75.0)	38 (69.1)		172 (83.5)	23 (51.1)	
Sometimes	205 (17.7)	31 (31.0)		177 (18.6)	11 (20.0)		28 (13.6)	20 (44.4)	
No	67 (5.8)	8 (8.0)		61 (6.4)	6 (10.9)		6 (2.9)	2 (4.4)	
Could discriminate between different types of stool			< 0.001			< 0.001			< 0.001
Yes	949 (81.9)	39 (39.0)		788 (82.7)	21 (38.2)		161 (78.2)	18 (40.0)	
Difficult	112 (9.7)	46 (46.0)		79 (8.3)	24 (43.6)		33 (16.0)	22 (48.9)	
No	98 (8.5)	15 (15.0)		86 (9.0)	10 (18.2)		12 (5.8)	5 (11.1)	
Could control bowels after urge sensation for:			< 0.001			< 0.001			< 0.001
< 1 min	90 (7.8)	35 (35.0)		67 (7.0)	17 (30.9)		23 (11.2)	18 (40.0)	
< 5 min	217 (18.7)	36 (36.0)		170 (17.8)	17 (30.9)		47 (22.8)	19 (42.2)	
< 15 min	226 (19.5)	8 (8.0)		183 (19.2)	7 (12.7)		43 (20.9)	1 (2.2)	
Never had to hurry	626 (54.0)	21 (21.0)		533 (55.9)	14 (25.5)		93 (45.1)	7 (15.6)	
Experiencing fecal urgency at least monthly	100 (8.6)	58 (58.0)	< 0.001	70 (7.4)	30 (54.5)	< 0.001	30 (14.6)	28 (62.2)	< 0.001
Abdominal pain at least monthly	234 (20.2)	48 (48.0)	< 0.001	181 (19.1)	26 (47.3)	< 0.001	53 (25.7)	22 (48.9)	0.008

Second, we analyzed the distribution of the following symptoms of FI: decreased ability to feel urge sensation before defecating, decreased ability to discriminate between different types of stool, the inability to control the bowels for a certain length of time after feeling urge sensation, experiencing fecal urgency at least once a month, and feeling abdominal pain at least once a month (Table 2). In the comorbidity subgroup, respondents who suffered from FI felt urge sensation before defecating significantly less often in comparison to fecally continent respondents (48.8 versus 16.5%, *P* < 0.001). In the “healthy” subgroup, we found no significant difference between respondents who did or did not suffer from FI regarding the feeling of urge sensation before defecating (30.9 versus 25.0%, *P* = 0.329). In all three groups, the respondents who suffered from FI were less able to discriminate between different stool types and experienced fecal urgency more often than the fecally continent respondents. Additionally, once the respondents with FI reached urge sensation, they were significantly less able to control their bowels for more than 5 min.

## Discussion

We demonstrated that in the general Dutch population the combination of soiling and urge FI was the most common subtype of FI, while passive FI was least common. Respondents in the comorbidity subgroup suffered from FI significantly more often and they suffered from a more severe form of FI than respondents in the “healthy” subgroup. The fact that respondents with comorbidities experienced FI was in itself not surprising. What was striking, however, was that the theoretically “healthy” group appeared not to be so healthy after all, considering the relatively high prevalence of FI in this subgroup. Apparently, FI occurs more often than thought, even in “healthy” subjects.

Why soiling and urge FI occur so often in the Dutch population is unknown, because the exact mechanisms underlying these subtypes of FI has not yet been determined. The high prevalence of soiling, however, may be caused by constipation, soiling is a known consequence of constipation. Recently, we demonstrated that the prevalence of constipation is as high as 24.5% in the general Dutch population [13]. Urge FI can be a result of a diminished conscious contraction of the external anal sphincter caused by pudendal neuropathy, which in turn may be caused by constipation, even though it may also be the result of factors such as pelvic surgery or somatic diseases.

The low prevalence of passive FI might be explained by our recent finding with regard to the underlying pathophysiology of passive FI [18, 19]. We found that passive FI can be the result of a diminished unconscious contraction of the external anal sphincter as seen in patients with a non-functioning anal-external sphincter continence reflex (AESCR). Furthermore, we found that unconscious contraction of the external anal sphincter is not affected by aging, in contrast to conscious contraction, which decreases with aging. [19] Thus, the elderly suffer from urge FI more often than from passive FI, thus explaining the low prevalence of passive FI in the Dutch population.

A few other studies also addressed the distribution of the subtypes of FI [7, 10, 11, 20]. In our opinion, however, our results cannot be compared with the results reported by these studies on account of differences in study methods. Most importantly, we included a random selection of the general population, while the other studies were limited to patients suffering from FI. Moreover, they used different definitions for the subtypes of FI.

We described the symptoms experienced by the respondents with FI. One of our findings in the comorbidity subgroup was that fecally incontinent respondents felt urge sensation before defecating less often than their fecally continent counterparts. This indicates that FI in the comorbidity subgroup might be caused by sensory nerve dysfunction on account of pelvic floor surgery or comorbidities such as diabetes mellitus. Our assumption is supported by an opposite observation in the “healthy” subgroup: respondents with FI felt urge sensation before defecating comparably often as fecally continent respondents.

Furthermore, we offer two explanations for nocturnal FI reported by 24% of the respondents in the comorbidity subgroup and a remarkable 7% in the “healthy” subgroup. First, in our clinical practice, we see patients with severe forms of dyssnergic defecation who suffer from nocturnal FI. We hypothesize that they, unintentionally, withhold their stool during the day. During sleep, however, conscious contraction to prevent unwanted loss of stool falls away, resulting in nocturnal FI. Second, we hypothesize that the anal-external sphincter continence reflex might be non-functional in some of these respondents. During their waking hours, patients with a non-functioning anal-external sphincter continence reflex are known to have trained themselves to respond to any rectal filling *sensation* by going to the toilet immediately, thus preventing FI. During asleep, however, this rescue system is inactive. Dysfunction of the anal-external sphincter continence reflex might be explained by nerve damage caused by surgery in the pelvic floor region or by comorbidities, such as diabetes mellitus.

Finally, we did not consider the obstetric history of women as a comorbidity because we recently showed that parity is not a risk factor for FI [21]. However, we did consider anal sphincter operation as a criteria to be assigned to the comorbidity subgroup. Furthermore, as demonstrated by Brusciano and others, it would be interesting to add clinical-functional measurements to demonstrate the underlying factors of FI in a follow-up study [22, 23].

### Limitations

This study might be biased as a result of the low response rate of 54%. This low rate may be explained by the nature of the topic and by the length of the questionnaire. We tried to limit response bias by creating a representative study group in which sex, region, and age were equally distributed in accordance with the population pyramid of the Netherlands. Furthermore, we formed the comorbidity subgroup on the basis of data reported by the respondents in the questionnaires and these data could not be validated against medical records because the questionnaires were anonymous. Anonymity was, however, helpful in obtaining honest answers to embarrassing questions on defecation habits.

### Conclusions

The majority of the general Dutch population who suffer from FI report the combination of soiling and urge FI, while the minority report passive FI. Respondents who suffer from chronic diseases or who underwent pelvic floor or bowel surgery suffer from more severe forms of fecal incontinence. In contrast to the “healthy” subgroup, respondents in the comorbidity subgroup who suffered from FI felt urge sensation before defecating significantly less often than their fecally continent counterparts. This indicates that FI in this group might be caused by sensory nerve dysfunction.

## Electronic supplementary material

ESM 1 (PDF 387 kb)
