# Supplementary material for: Subtypes and symptoms of fecal incontinence in the Dutch population: a cross-sectional study
Source: Int J Colorectal Dis. 2018 Apr 28;33(7):919–25. doi: 10.1007/s00384-018-3051-5 (PMC6002467; doi:10.1007/s00384-018-3051-5)
Supplement: Supplementary file 1 — (PDF 387 kb) [file 384_2018_3051_MOESM1_ESM.pdf]

The Groningen Defaecation & Faecal Continence Checklist

Instructions:

1. Answer the questions by ticking the box next to your answer. Please tick just one answer to each question (unless you are invited to give more than one answer).
2. Although some of the questions may seem very similar, each one gives us important information. Some of the questions might relate to problems you do not have, but we want to know this too. Please answer every question (unless you are specifically told to proceed to another question).
3. There are no right or wrong answers. If you are unsure about how to answer a question, try to choose the answer that comes closest to your situation.
4. If you have any comments about the questionnaire, or if there is anything else you would like to say but which has not been covered by the questions, you can add your own comments at the end of the questionnaire.
5. Your answers will be treated in the strictest confidence.

umcg

Personal details

Surname ______________________

First name ______________________

Date of birth ______________________

Height (cm) ______________________

Weight (kg) ______________________

0.1 What is your gender?

- ☐ Male
- ☐ Female

0.2 What is your age in years?

____

0.3 In which province do you live?

☐ Drenthe	☐ Noord-Brabant
☐ Flevoland	☐ Noord-Holland
☐ Friesland	☐ Overijssel
☐ Gelderland	☐ Utrecht
☐ Groningen	☐ Zeeland
☐ Limburg	☐ Zuid-Holland

0.4 How big is the town or village in which you live?

- ☐ I live in a village
- ☐ I live in a small town with fewer than 50,000 inhabitants
- ☐ I live in a medium-sized town with 50,000 to 100,000 inhabitants
- ☐ I live in a large town with more than 100,000 inhabitants

0.5 What is your highest level of education?

- ☐ Primary school education
- ☐ Level 1 or 2 BTEC or equivalent vocational qualification
- ☐ GCSEs with fewer than 5 grade A*-C or equivalent
- ☐ Level 3 or 4 BTEC or equivalent vocational qualification / apprenticeship
- ☐ 5+ GCSEs grade A*-C or equivalent
- ☐ 3+ A-Levels or equivalent
- ☐ Level 5 BTEC or equivalent vocational qualification / Foundation Degree
- ☐ University education
- ☐ Other, namely: ____________________

0.6 What is/was your job or profession?

____

0.7 Are you still working?

- ☐ Yes, I work __________ hours per week
- ☐ No, I am no longer in paid employment, because:
- ☐ I spend my time doing housework and/or looking after the children
- ☐ I am retired or have taken early retirement
- ☐ I am at school, college or university
- ☐ I do not have a paid job due to problems with my bowels and/or pelvic floor
- ☐ I do not have a paid job due to other health problems
- ☐ I do not have a paid job for other reasons (e.g. I cannot find one, I do voluntary work, etc.)

0.8 In general, how would you describe your health in relation to the ability to hold and pass stools?

- ☐ Very good
- ☐ Good
- ☐ Reasonable
- ☐ Poor
- ☐ Very poor

Category 1: Defecation pattern

The following questions refer to your defecation pattern over <u>the past six months</u>.

1.1 On average, how often do you empty your bowels? (**Only tick one box**)

- ☐ Less than once a month
- ☐ Less than once a week
- ☐ Once a week
- ☐ Twice a week
- ☐ Once every two days
- ☐ Once or twice a day
- ☐ Three to five times a day
- ☐ More than five times a day

1.2 In general, what did your faeces look like (which type do you have most often)? (**Only tick one box**)

☐ 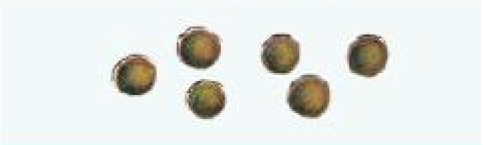 Separate hard lumps (hard to pass)

☐ 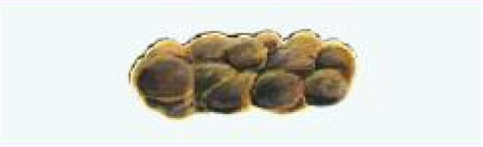 Sausage-shaped but lumpy

☐ 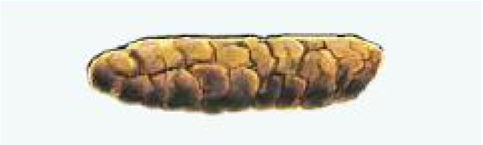 Like a sausage but with cracks on its surface

☐ 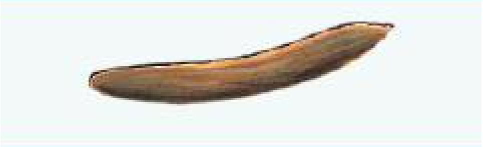 Like a sausage or snake, smooth and soft

☐ 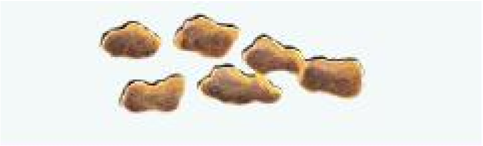 Soft blobs with clear-cut edges (passed easily)

☐ 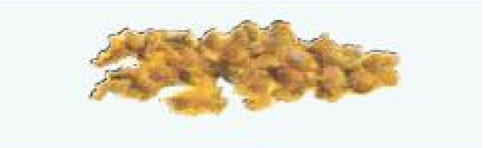 Fluffy pieces with ragged edges, a mushy stool

☐ 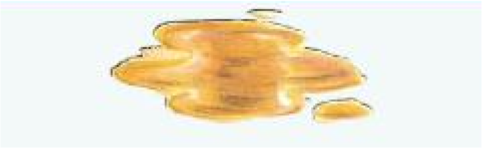 Watery, no solid pieces (enterily liquid)

Category 2: Constipation

The following questions are about the difficulty you have had emptying your bowels over <u>the past six months</u>.

2.1 Did you have difficulty emptying your bowels (e.g. because of hard stools, not being able to pass all your stools or having to strain hard)?

- ☐ Yes
- ☐ No

2.1.1 If so, how long have you had this problem?

- ☐ 0-1 year
- ☐ 1 to 5 years
- ☐ 5 to 10 years
- ☐ 10 to 20 years
- ☐ Longer than 20 years

2.2 How often did you have to strain hard to empty your bowels?

- ☐ Never
- ☐ Less than once a month
- ☐ Several times a month
- ☐ Several times a week
- ☐ Every day

2.3 On average, how long did you have to strain while emptying your bowels?

- ☐ Less than 5 minutes
- ☐ 5 to 10 minutes
- ☐ 10 to 20 minutes
- ☐ 20 to 30 minutes
- ☐ Longer than 30 minutes

2.4 How often did you have trouble passing stools because it felt as if there was a blockage?

- ☐ Never
- ☐ Less than once a month
- ☐ Several times a month
- ☐ Several times a week
- ☐ Every day

2.5 How often did it feel as if you had not completely emptied your bowels after passing stools?

- ☐ Never
- ☐ Less than once a month
- ☐ Several times a month
- ☐ Several times a week
- ☐ Every day

2.6 How often did you manage not to pass stools after feeling the urge to empty your bowels?

- ☐ I always manage
- ☐ One to three times a day
- ☐ Four to six times a day
- ☐ Seven to nine times a day
- ☐ More than nine times a day

2.7 How often did you have to return to the toilet within one hour of emptying your bowels to empty them again?

- ☐ Never
- ☐ Less than once a month
- ☐ Several times a month
- ☐ Several times a week
- ☐ Every day

2.8 How often did you have pain in your anus while emptying your bowels?

- ☐ Never
- ☐ Less than once a month
- ☐ Several times a month
- ☐ Several times a week
- ☐ Every day

2.9 Have you suffered from abdominal bloating?

- ☐ Yes
- ☐ No

2.9.1 If so, to what extent? (You may tick more than one answer)

- ☐ I only felt it myself
- ☐ Other people could also see it
- ☐ It made me lose my appetite or feel sick
- ☐ It made me vomit

2.10 How often did you have abdominal pain or cramps?

- ☐ Never
- ☐ Less than once a month
- ☐ Several times a month
- ☐ Several times a week
- ☐ Every day

If you did not experience abdominal pain or cramps during the past six months, please proceed to question 3.1.

2.10.1 If you did experience abdominal pain or cramps, was this only during your menstrual period?

- ☐ No
- ☐ Yes
- ☐ Not applicable because I am post-menopausal
- ☐ Not applicable because I am a man

2.10.2 If you did experience abdominal pain or cramps, did they disappear or recede after you had emptied your bowels?

- ☐ Never or rarely
- ☐ Sometimes
- ☐ Often
- ☐ Usually
- ☐ Always

2.10.3 Do you have go to the toilet to empty your bowels more or less frequently since the abdominal pain or cramps started?

- ☐ Yes, I go to the toilet more frequently than before
- ☐ Yes, I go to the toilet less frequently than before
- ☐ No, I go to the toilet just as often as before

2.10.4 Has the consistency of your stools changed since the abdominal pain or cramps started? (Have they become harder or softer, for example)

- ☐ Yes, my stools are harder
- ☐ Yes, my stools are softer
- ☐ No, the consistency has not changed

Category 3: Constipation-related questions

The following questions relate to your diet and any remedies you may have used to help you empty your bowels during <u>the past six months</u>.

3.1 Do you drink at least 1.5 litres of fluids a day (10 x 150ml-cups/glasses)?

- ☐ Yes
- ☐ No

3.2 Do you eat at least 2 pieces of fruit a day?

- ☐ Yes
- ☐ No

3.3 Do you eat at least 3 tablespoons of vegetables a day?

- ☐ Yes
- ☐ No

3.4 Do you eat at least 3 slices of brown or wholemeal bread a day?

- ☐ Yes
- ☐ No

3.5 How often do you take laxatives to soften your stools/make it easier to empty your bowels?

- ☐ Never
- ☐ Less than once a month
- ☐ Several times a month
- ☐ Several times a week
- ☐ Once a day
- ☐ Several times a day

3.5.1 If you take laxatives, which one do you take and how much?

1. Medicine: ________________ How often per day: _____ Dosage: ____ ml/g

Or per week: _____

2. Medicine: ________________ How often per day: _____ Dosage: ____ ml/g

Or per week: _____

3. Medicine: ________________ How often per day: _____ Dosage: ____ ml/g

Or per week: _____

3.6 Do you eat a special diet or foods to soften your stools?

☐ Yes, I eat /drink: ______________________

☐ No

3.7 Do you use an enema (= injecting a small amount of a medicine into the anus) to help pass stools?

☐ Yes, medicine: ______________________ dosage: _____ ml/cc

☐ No

3.7.1 If so, how often?

☐ Less than once a month

☐ Several times a month

☐ Several times a week

☐ Once a day

☐ Several times a day

3.8 Do you irrigate your rectum with lukewarm water (via the anus or by means of an antegrade colonic enema) to help you empty your bowels?

- ☐ Yes, amount: _____ ml/cc, with (if applicable): ______________
- ☐ No

3.8.1 If so, how often did you irrigate?

- ☐ Less than once a month
- ☐ Several times a month
- ☐ Several times a week
- ☐ Once a day
- ☐ Several times a day

3.9 Do you ever use your fingers or hands to help pass stools? (You may tick more than one answer)

- ☐ Yes, I press on my abdomen with my hands
- ☐ Yes, I use my finger to press between my buttocks, just <u>in front of</u> the anus
- ☐ Yes, I use my finger to press between my buttocks, just <u>behind</u> the anus
- ☐ Yes, I use my fingers to remove stools from my anus
- ☐ Yes, but in another way, namely: ______________________
- ☐ No

3.9.1 If so, how often do you use your fingers or hands when passing stools?

- ☐ Less than once a month
- ☐ Several times a month
- ☐ Several times a week
- ☐ Every day

3.10 If you had difficulty passing stools, have you ever talked to anyone about it? (You may tick more than one answer)

- ☐ Not applicable, I do not have difficulty passing stools
- ☐ Yes, with family or friends
- ☐ Yes, with my GP
- ☐ Yes, with a medical specialist
- ☐ Yes, with someone else, namely: ____________________
- ☐ No

Category 4: Faecal continence

The following questions are about the accidental passage of stools during the past six months.

4.1 How often did you accidentally pass small amounts of faeces? (i.e. stained/soiled your underpants)

- ☐ Never
- ☐ Less than once a month
- ☐ Several times a month
- ☐ Several times a week
- ☐ Once a day
- ☐ Several times a day

4.1.1 If you accidentally passed small amounts of faeces, when did this happen? (You may tick more than one answer)

- ☐ When I had diarrhoea
- ☐ When I was desperate for the toilet
- ☐ During physical activity/exertion
- ☐ For no clear reason

4.2 How often did you accidentally pass large amounts of solid faeces without having felt an urge (i.e. without feeling the need for the toilet)?

- ☐ Never
- ☐ Less than once a month
- ☐ Several times a month
- ☐ Several times a week
- ☐ Once a day
- ☐ Several times a day

4.3 How often did you feel a strong urge to empty your bowels but were unable to reach the toilet in time?

- ☐ Never
- ☐ Less than once a month
- ☐ Several times a month
- ☐ Several times a week
- ☐ Once a day
- ☐ Several times a day

4.4 How often did you accidentally pass watery stools (diarrhoea)?

- ☐ Never
- ☐ Less than once a month
- ☐ Several times a month
- ☐ Several times a week
- ☐ Once a day
- ☐ Several times a day

4.5 How often did you accidentally pass wind?

- ☐ Never
- ☐ Less than once a month
- ☐ Several times a month
- ☐ Several times a week
- ☐ Once a day
- ☐ Several times a day

If you have not accidentally passed liquid or solid stools during the past six months, please proceed to question 5.1.

4.6 If you have accidentally passed faeces, how much was this on average?

- ☐ A tiny amount, about the size of a coin
- ☐ Enough to make me change my underpants
- ☐ Enough to make me change my underpants and trousers

4.7 If you accidentally passed faeces, when did this happen?

- ☐ Only while I was awake
- ☐ Only while I was asleep
- ☐ While I was awake and while I was asleep

4.8 How often did you use panty liners or incontinence pads to help when you accidentally passed faeces?

- ☐ Never
- ☐ Less than once a month
- ☐ Several times a month
- ☐ Several times a week
- ☐ Once a day
- ☐ Several times a day

4.9 How often did you rearrange your daily programme because of accidentally passing faeces (e.g. stayed at home, cancelled an appointment, changed your diet)?

- ☐ Never
- ☐ Less than once a month
- ☐ Several times a month
- ☐ Several times a week
- ☐ Once a day
- ☐ Several times a day

4.10 Have you ever accidentally passed faeces shortly after emptying your bowels on the toilet?

- ☐ Yes
- ☐ No

4.11 Do you use an anti-diarrhoea medicine to solidify your stools?

- ☐ Never
- ☐ Less than once a month
- ☐ Several times a month
- ☐ Several times a week
- ☐ Once a day
- ☐ Several times a day

4.11.1 If you use an anti-diarrhoea medicine, which one do you use and how much?

1. Medicine: ________________ How often per day: ____ Dosage: ____ ml/g
Or per week: ____

2. Medicine: ________________ How often per day: ____ Dosage: ____ ml/g
Or per week: ____

3. Medicine: ________________ How often per day: ____ Dosage: ____ ml/g
Or per week: ____

4.12 Do you eat a diet or eat particular foods to control accidental passage of stools?

- ☐ Yes, I eat/drink: _____________________
- ☐ No

4.13 Do you irrigate your bowels with lukewarm water to control accidental passage of stools?

- ☐ Yes, amount: _____ ml/cc, with (if applicable): ______________
- ☐ No

4.14 Have you ever talked to anyone about losing control of your bowels? (You may tick more than one answer)

- ☐ Yes, with family or friends
- ☐ Yes, with my GP
- ☐ Yes, with a medical specialist
- ☐ Yes, with someone else, namely:

Category 5: Urge

The following questions are about your urge to go the toilet over <u>the past six months</u>.

5.1 Did you feel the urge to empty your bowels before you went to the toilet?

- ☐ Yes
- ☐ Sometimes
- ☐ No

5.2 On average, how long were you able to control your bowels once you had felt the urge to go to the toilet?

- ☐ I was unable to control my bowels
- ☐ One minute or less (I always had to go to the toilet immediately)
- ☐ Five minutes at the most
- ☐ Fifteen minutes at the most
- ☐ I never had to hurry

5.3 How often did you have to hurry to get to the toilet in time, to prevent yourself accidentally passing stools?

- ☐ Never
- ☐ Less than once a month
- ☐ Several times a month
- ☐ Several times a week
- ☐ Once a day

5.4 When you felt the urge to go to the toilet, could you tell the difference between flatulence, diarrhoea and solid/hard stools?

- ☐ Yes
- ☐ With difficulty
- ☐ No

Category 6: Urinary incontinence

The following questions concern bladder control over <u>the past six months</u>.

6.1 On average, how often did you urinate?

- ☐ Less than three times a day
- ☐ Three to seven times a day
- ☐ More than seven times a day

6.2 When you urinated, were you able to empty your bladder in one go?

- ☐ Yes, the urine stream was <u>never</u> interrupted
- ☐ No, the urine <u>sometimes</u> came in bursts (stopped and started)
- ☐ No, the urine <u>always</u> came in bursts (stopped and started)

6.3 When you urinated, did you have to strain?

- ☐ Yes, I always had to strain while urinating
- ☐ Yes, I sometimes had to strain while urinating
- ☐ No, I never had to strain while urinating

6.4 How often did you accidentally lose urine?

- ☐ Never
- ☐ About once a week or less
- ☐ Two to three times a week
- ☐ About once a day
- ☐ Several times a day
- ☐ Continuously

6.5 How much urine did you lose on average (irrespective of whether you used pads)?

- ☐ None
- ☐ A bit (a few drops)
- ☐ Quite a lot (wet underpants)
- ☐ A lot (visible wet patches)

6.6 When did you accidentally lose urine? (You may tick more than one answer)

- ☐ Never, I did not lose any urine
- ☐ Before I could reach the toilet
- ☐ Whenever I sneezed or coughed
- ☐ While I was asleep
- ☐ During physical activity/exertion
- ☐ When I got dressed again after urinating
- ☐ For no clear reason
- ☐ Continuously

6.7 How often did you need to go to the toilet during the night?

- ☐ Never/rarely
- ☐ Once or twice a week
- ☐ Three to six times a week
- ☐ Every night
- ☐ Several times a night

6.8 How often did you feel as if you had a bladder infection in the past 6 months?

- ☐ Never
- ☐ Once
- ☐ Several times

6.9 How often have you been treated for a bladder infection in the past 6 months?

- ☐ Never
- ☐ Once
- ☐ Several times

Category 7: Obstetric and gynaecological history

The following questions only apply to women. If you are a man, please proceed to question 8.1.

7.1 Have you ever been through childbirth (including caesarean section)?

☐ Yes

☐ No

7.1.1 If so, how many times?

____

7.2 How many of these were natural (vaginal) deliveries?

____

If you have never experienced a vaginal delivery, please proceed to question 7.7.

7.3 How long did you have to push during your longest delivery?

☐ Less than one hour

☐ One to two hours

☐ Longer than two hours

7.4 Were obstetrical instruments used during any of these vaginal deliveries?

☐ Yes

☐ No

7.4.1 If so, which instruments were used? (You may tick more than one answer)

Forceps

☐ A vacuum extractor

☐ Other, namely

7.5 Did you need an incision in the perineum (episiotomy) or did you rupture during a vaginal delivery, to the extent that the pelvic floor muscles around your anus were affected?

☐ Yes

☐ No

7.5.1 If so, what happened? (You may tick more than one answer)

☐ I ruptured

☐ I had an incision in the perineum (episiotomy)

☐ Other, namely ____________________

7.6 What was the weight of your **heaviest** baby?

__________ grams

7.7 Has your uterus been removed (a hysterectomy)?

☐ Yes, via the vagina (vaginal)

☐ Yes, via the abdomen (abdominal)

☐ No

7.8 When you are emptying your bowels, does it ever feel as if something is hanging out or descending through your vagina?

☐ Yes

☐ No

Category 8: Medical history

The following questions relate to conditions or operations that may affect your bowel control.

8.1 Have you ever undergone one of the following surgical procedures that may affect your bowel control? (You may tick more than one answer)

- ☐ No, I have never had an operation on my bowels, anus or prostate
- ☐ Removal of a section of bowel, after which the remaining sections were sutured together
- ☐ Operation on a fistula in the anal cleft close to the anus (perianal fistula)
- ☐ Operation on the anal sphincter
- ☐ Operation for haemorrhoids
- ☐ Operation on the prostate
- ☐ Other, namely: ____________________

Procedure to repair a hereditary condition, such as:

- ☐ Anal atresia or congenital anorectal malformation
- ☐ Hirschsprung’s disease
- ☐ Sacrococcygeal teratoma

8.2 Do you have (or have you had) a stoma to remove faeces from your bowel?

- ☐ Yes, a colostomy
- ☐ Yes, an ileostomy
- ☐ No

8.3 Do you ever have blood and/or mucous in your stools?

- ☐ Yes
- ☐ No

8.4 Have you ever had an injury to your anus, apart from during childbirth or an operation?

- ☐ Yes, namely: ____________________
- ☐ No

8.5 Have you ever had, or are you still experiencing the after-effects of, one of the following medical conditions? (You may tick more than one answer)

- ☐ I have never had any of the conditions listed below
- ☐ Crohn’s disease or colitis ulcerosa (inflammation of the colon)
- ☐ Irritable bowel syndrome
- ☐ Prolapse of the rectum
- ☐ Diabetes mellitus
- ☐ Cerebral haemorrhage or infarction (stroke)
- ☐ Another neurological conditions (e.g. paraplegia, multiple sclerosis)
- ☐ Slow transit constipation

Hereditary conditions such as:

- ☐ Anal atresia or congenital anorectal malformation
- ☐ Hirschsprung’s disease
- ☐ Sacrococcygeal syndrome
- ☐ Spina bifida
- ☐ Other, namely: ____________________

8.6 Does one of the medical conditions you have ticked occur in your family?

- ☐ Yes
- ☐ No
- ☐ Not applicable

8.6.1 If so, which conditions occur in which members of your family?

Condition: ____________________ Relative: ____________________

Condition: ____________________ Relative: ____________________

Condition: ____________________ Relative: ____________________

Condition: ____________________ Relative: ____________________

umcg

8.7 Which medicines do you take at the moment (you do not need to mention the laxatives and anti-diarrhoea treatments mentioned previously)?

☐ I do not take any other medication.

☐ I take:

1. Medicine: ____________________ How often per day: _____ Dosage _____ ml/g
2. Medicine: ____________________ How often per day: _____ Dosage _____ ml/g
3. Medicine: ____________________ How often per day: _____ Dosage _____ ml/g
4. Medicine: ____________________ How often per day: _____ Dosage _____ ml/g
5. Medicine: ____________________ How often per day: _____ Dosage _____ ml/g
6. Medicine: ____________________ How often per day: _____ Dosage _____ ml/g

You have come to the end of the questionnaire.

Thank you very much for taking the time to answer these questions.

If there is anything else you would like to say, or if there is something you feel was not covered or not covered sufficiently by this questionnaire, please use the space below to leave your comments.
